# Use of a Modified DANP-mV Model to Improve Quality of Life in Rural Residents: The Empirical Case of Xingshisi Village, China

**DOI:** 10.3390/ijerph16010153

**Published:** 2019-01-08

**Authors:** Guang-Bin Qu, Tian-Yu Zhao, Bo-Wei Zhu, Gwo-Hshiung Tzeng, Shan-Lin Huang

**Affiliations:** 1 Key Laboratory of Cold Region Urban and Rural Human Settlement Environment Science and Technology, Ministry of Industry and Information Technology, School of Architecture, Harbin Institute of Technology, 92 West Dazhi Street, Nan Gang District, Harbin 150006, China; sharif76@sina.com (G.-B.Q.); 13904658044@139.com (T.-Y.Z.); 2 Graduate Institute of Urban Planning, College of Public Affairs, National Taipei University, 151, University Rd., San Shia District, New Taipei City 23741, Taiwan; zhubowei301@gmail.com (B.-W.Z.); ghtzeng@gm.ntpu.edu.tw (G.-H.T.); 3 Faculty of Humanities and Arts, Macau University of Science and Technology, Avenida Wai Long, Taipa 999078, Macau, China; 4 Department of Tourism Management, Tourism School, Sanming University, 25, Jingdong Rd., Sanyuan District, Sanming 365004, China; 5 National Park Center, Sanming University, 25, Jingdong Rd., Sanyuan District, Sanming 365004, China

**Keywords:** village livability, quality of life, WHOQOL-BREF, DANP-mV model, aspiration level, benchmark

## Abstract

Climate change-related anomalies have increased public concern regarding environmental protection. This has opened newer rural development avenues. In this regard, livability of villages is crucial; it can be evaluated based on the villagers’ quality of life (QoL). The WHOQOL-BREF, a comprehensive cross-cultural and cross-disciplinary scale proposed by the World Health Organization to assess QoL, has aided in assessing and improving QoL in different regions. However, the factors of this instrument are mutually influential, necessitating an improvement strategy considering the entire system. This problem may be resolved using the DANP-mV model. However, the traditional DANP-mV model includes many items and responding to all of them is difficult for experts. Therefore, by using the case of Xingshisi Village in China, this study proposed a modified DANP-mV model to provide additional suggestions for systematic improvement of the QoL and livability in the village. Xingshisi is a model village built according to an aspirational benchmark; however, different from the traditional definition of a benchmark, this village exhibits room for improvement. Although the modified model reduces the number of questions from 650 to 168, its effect remains similar to that of the traditional model. Moreover, in the modified model, physical capacity (*D*_1_) presented the largest dimensional gap. The interaction among the factors indicated that considering the effect of the environment (*D*_4_) and developing a systematic improvement strategy are necessary to improve the livability of villages facing limited resources.

## 1. Introduction

Cities are akin to organisms in that they can grow or shrink at any time. The process of urbanization can be compared to magnetization; a city continuously attracts resources from neighboring regions and these resources combine and interact, generating momentum that prompts the city to sprawl out and expand [1]. Eventually, a megacity is born [2]. By contrast, absorbed regions decline because of population and industry migration. In addition to second-tier cities, the rural region also suffers from such changes, therefore, scholars have directed their attention to these declining regions [3,4,5]. Some scholars have suggested urban regeneration in second-tier cities through the revitalization of culture or heritage sites [4]. Other scholars have adopted the logic of revitalization–regeneration of the rural region [5,6]. They propose revitalizing the region by discovering and marketing local features that can be used to attract tourists [3]. However, some scholars have questioned whether rural regions can still be called “rural” following such development [7]. These scholars have claimed that development should be focused on the essence of rural area instead of on superficial elements. That is, these scholars are more concerned with the life of local residents. Enhancing the quality of life (QoL) of local residents is the primary agenda of these scholars [8,9]. 

The World Health Organization (WHO) proposed a framework of indices for QoL assessment called WHOQOL-BREF [10,11,12]. This set of indices provides a comprehensive and cross-cultural framework from multiple aspects to assess the happiness status of people in a specific region, such as Hong Kong, Korea, and China [13,14,15,16]. Many scholars have verified the appropriateness of this instrument or applied it to improve the QoL in a region. However, the factors of this instrument are mutually influential. The DANP-mV model is a suitable for resolving this problem [17]. This model uses a hybrid multiple-criteria decision-making (MCDM) method and is composed of two methods: DEMATEL based-ANP (DANP) and modified VIKOR (VIšekriterijumsko KOmpromisno Rangiranje) [18]. The DANP technique can generate influential network relation map (INRM) and influential weights (IWs). The relationship between each factor can be shown in the INRM [19]. IWs can identify the priority of each factor [20]. This model can also calculate the gap from the aspiration levels for each alternative through the part involving the modified VIKOR technique to further sort all the alternatives [21].

During China’s rapid rise to become an economic superpower, major cities in various provinces were involved in intense competition to effectively elevate their status in the tier system of cities and secure resources. Hence, local governments have consciously engaged in or accelerated urbanization to maintain high regional economic development. China’s central government continues to consider the improvement and development of rural area. On the basis of the everyday life perspective, the government proposed the vision of a “Beautiful Countryside”, hoping to create a new type of urban and rural development. This study mainly investigated the QoL of residents in Xingshisi Village, a rural village in northeast China. Continually developed and renovated according to the ideal of beautiful countryside, this village has achieved a high QoL, serving as a benchmark in northeastern China. However, are all benchmarks impeccable? Does benchmarks need to be improved [18]? The users of DANP-mV model believe that the benchmark must be continuously improved to achieve sustainable development [19]. Systematically, this model emphasizes that real improvements can only be attained by exploring the sources of influence. Without considering the overall system, problems cannot be addressed fully [20]. Thus, decision-makers can propose a fundamental strategy of supporting measures to determine methods for helping cases achieve ideal benchmarks by using this model [21,22]. Although this model has numerous advantages, its excessive number of items has always been a drawback. Because this model is based on expert experience, completing the questionnaire may not be difficult for the experts, but excessive items can cause some inconvenience. 

This study resolved this drawback by employing the modified DANP-mV model to explore how model villages such as Xingshisi Village systematically track and improve root of the problem and move toward the goal of increased livability. Whether the modified DANP-mV model can effectively reduce the number of questions while maintaining nearly the same effect to address the limitations of the old model was also determined. Mutually supportive strategies for improving from a system-level viewpoint to enhance the QoL in rural areas and aid rural residents in improving livability were identified. To achieve this, this study had three objectives: (1) find the problem (i.e., analyze the current QoL in the village and determine the problem), (2) develop the modified DANP-mV model (i.e., this model can significantly reduce the number of required items while having almost the same effect), and (3) provide suggestions for continuous improvement of Xingshisi Village (i.e., a rural community in northeastern China, where Chinese is the common language). Therefore, this study, based on the framework of the Chinese version of WHOQOL-BREF, proposes systematic improvement suggestions for QoL in Xingshisi Village by using the new model. 

The remaining paper is organized as follows: Section 2 reviews the indicator framework of the WHOQOL-BREF to determine the evaluation attributes for QoL in rural areas and presents the reasons for using the DANP-mV model and the pretest of indicators. Section 3 describes the innovations of the DANP-mV model, differences between the traditional and modified DANP-mV models, and procedure of the modified DANP-mV model. Section 4 describes case selection and presentation, data collection and analysis, and presents some discussion. Section 5 focuses on the crucial findings and discusses future research directions.

## 2. Research Framework Based on WHOQOL-BREF

The WHOQOL-BREF framework, the main theoretical basis used in this study, is first explained here. In addition, we discuss the impact relationships between various indicators to illustrate the appropriateness of the DANP-mV model. Finally, the framework for local heterogeneity considered. So this section is divided into three sections: Section 2.1. The framework of WHOQOL-BREF; Section 2.2. Reasoning for using the DANP-mV model; Section 2.3. Modifying the indicator framework after pretesting rural residents.

### 2.1. The Framework of WHOQOL-BREF

The WHOQOL-BREF is a powerful wellness index developed by the WHO after consulting international experts in related fields through a series of discussions [23,24,25]. Its purpose is to measure the QoL for residents in any area [24]. They can be classified into four dimensions: physical capacity, psychological state, social relationships, and environment [25]; after further expanding four dimensions in 1995, the WHO revised it to become the WHOQOL-100 [26]. The WHOQOL-100 can be classified into six dimensions: physical capacity, psychological state, social relationships, environment, independence, and spirituality [27,28]. However, more than 100 items are present in the index. Excessive numbers of items cause problem for questionnaire respondents and pose difficulties in gathering data. This is a notable obstacle for those using this tool to measure [29]. Thus, the WHOQOL-BREF was derived from the WHOQOL-100, and the original independence and spirituality facets were integrated under psychological factors [30]. Finally, the current version has 26 items with two additional items from the overall QoL and general health dimensions [31].

The likelihood of physical illness and diseases often increases with age. Experts who initially devised the WHOQOL-BREF used the physical capacity dimension as a basis for measuring QoL [32]. In addition to aging, the main cause of daily-life obstacles is disease occurrence. Thus, when assessing physical capacity, it is necessary to consider “whether or not a disease exists”, “pain and discomfort”, and “dependence on medical substances and medical aids” as the basis of measurement [33]. The second step is the investigation of “action capabilities,” which include the criteria of “energy and fatigue,” “work capacity,” “sleep and rest,” “activities of daily living,” and “mobility” as the foundation for measurement [34]. The criteria for energy and fatigue are measured by the energy of the respondent’s daily life or energy for daily life. Work capacity is a measurement of work status, and sleep and rest refers to the sleep quality of the respondent. Activities of daily living are necessary activities to test whether the respondent can cope with daily life, and mobility refers to the ability of respondents to move and get around [35].

Chronic diseases require continual treatment. Patients with chronic diseases not only have from long-term physical problems but also endure effects on their spirit, including their psychological state, potentially ultimately leading to a decline in overall QoL [29,36,37]. Consequently, assessment of the psychological dimension is also crucial. The first step is to explore the values of respondents in everyday life when faced with daily life problems. First, we explore the values of respondents using items such as negative and positive feelings as indicators [38]. The second step is to understand the subject’s self-knowledge by using self-esteem and body image” as evaluation indicators. The last step is to measure the mental state of the subjects and whether they have faith and spiritual sustenance with “spirituality, religion, personal beliefs, thinking, and learning” and “memory and concentration” as criteria [39].

When interacting with others, people can share a certain amount of happiness by sharing or releasing pressure through narration. Based on this, we know that “interaction with others” is an factor influencing QoL [40]. The WHOQOL-BREF dimension used to measure this interaction is “social relationship”. It considers interactions between relatives, family, and friends and uses “personal relationships” and “social support” as indicators. In particular, the WHO believes that sex is a crucial factor in maintaining QoL [41]. Therefore, WHOQOL-BREF uses “sexual activity” as an evaluation factor. In the Chinese world, the factor of “respect by others” for the respondents is also expected to affect QoL. Thus, this study added this factor to explore [16].

QoL and performance scores in numerous dimensions decrease with age. When people become older, they rely more on the support provided by their environment [42]. Therefore, the dimension of environment in WHOQOL-BREF includes eight indicators. The first explores a subject’s daily life space and classifies it into “home environment”. Second, “physical environment” represents the extent to which residents in an area withstand pollution. The degree of pollution here is based on a broad concept; in addition to air and water pollution, noise pollution and pollution caused by the public transportation system are also considered. The third indicator is “freedom, physical safety, and security”. This indicator is used to measure freedom and safety of living spaces [43]. The other five criteria assess the degree to which the environment provides sufficient living function for residents [41,42,43,44]. First, the richness of medical resources is a crucial factor affecting the QoL of residents in the region. Easy access to medical resources is vital for elderly people. The WHOQOL-BREF uses the indicator “health and social care: accessibility and quality”. The second indicator is “participation in and opportunities for recreation or leisure activities”. Leisure is a relaxing activity. The environment of an area must provide suitable places for residents to relax. This has a positive effect on the maintenance of QoL. The third indicator is “opportunities for acquiring new information and skills”. This indicator measures the availability of information in an area. Information here uses a wide interpretation; in addition to information about life, it includes information regarding education and learning. The fourth indicator is “financial resources”. In addition to finances earned, financial resources here refer more to whether the area has a pipeline for residents of the region to accumulate wealth. Finally, the “transport” indicator is used to measure mainly the quality of the transportation system, which refers to whether residents in the area can easily reach their final destination via the public transportation system. Moreover, the “convenience of dining” is used as an indicator to investigate the convenience of the living environment for eating and drinking. 

### 2.2. Reasoning for Using the DANP-mV Model

The WHOQOL-BREF examines the QoL of residents in a specific region from the aspects of physical capacity, psychological state, social relationships, and the environment, which tend to be correlated [36,45]. For example, scholars have conducted thorough research on patients with chronic diseases such as AIDS [37]. Their results reveal that because the treatment of chronic diseases is often a prolonged process and from which full recovery cannot be guaranteed, patients with these diseases must face social pressure in addition to bearing the chronic physical pain and discomfort. Such a situation can generate stress in the patients, further leading to the deterioration of their overall QoL. If patients receive some support, regardless of whether it is substantial support provided by their overall environment, the tolerance of society, or spiritual beliefs held by the patients, such support can have a positive effect on their QoL [8,46]. In research on the QoL of patients with urinary incontinence, notable changes were found in the psychological and physical states as well as the social relationships of patients who received medical treatments. This shows that in the real world, factors interact to jointly influence QoL. Thus, a method that can loosen the independence of independent variables is warranted [23,24,25]. 

The DANP-mV model is a method for conducting performance evaluation and proposing improvement strategies in a situation where mutually influencing factors are present. A previous study on the design of public open spaces indicated that mobility, natural enjoyment, and social interaction must be considered for the designed space to be effective in promoting healthy aging [47]. From the results of the examined case, the scholars determined that the performance in natural enjoyment, or the sense of enjoyment derived from being immersed in the natural environment, was the poorest. Moreover, from studying the criteria in the dimension of natural enjoyment, they found that the major problem was a lack of water features. In this case, if this study were conducted under the assumption of independence, the improvement method would have been to add a wetland or a fountain. However, because this case was located at high latitude, the maintenance of wetlands or fountains would have become another source of problems under the cold climate. Therefore, whether such arrangements could facilitate enhancing natural enjoyment would become debatable. Hence, the authors of that study proposed suggestions for strategy improvement by considering the source of the problem (i.e., high-quality vegetation). Thus, the authors concluded that to effectively promote healthy aging in that region, resources should be invested in increasing high-quality vegetation. In another study, a case study on the economic sustainability of Taichung Cultural and Creative Industry Park showed that to actually improve the economic state of that park and ensure economic sustainability, information transmission within the creative community must be strengthened and communication channels between institutions and the creative community must remain open [48]. The DANP-mV model has been applied to the research of numerous fields [49]. Proposals of improvement strategies considering the sources of influence have been observed in studies on how to achieve a better life and how to mitigate China’s risk in cloud computing.

### 2.3. Modifying the Indicator Framework after Pretesting Rural Residents

The WHOQOL-100 is a cross-cultural framework of indices, the results of which are comparable across cultures [50]. It was developed by the WHO for QoL measurement in which the definitions of a satisfactory life in different cultures are compiled. The WHOQOL-BREF is a shorter version of the WHOQOL-100 developed for practical applications; therefore, the WHOQOL-BREF also exhibits interdisciplinary and cross-cultural characteristics [41]. Numerous scholars have tested the applicability of this scale in various cases. These studies have mostly investigated how to clearly convey the meaning in the original scale in the translated versions. Some have involved screening collected data using statistical methods that determine the reliability and validity of the data and the eventual presentation of the data [14,51].

The pursuit of a high QoL is not a concern restricted to urban residents. Rural residents also have this need [8,9]. However, rural and urban area are fundamentally different. Urban residents may aspire for more green spaces, but in rural area where green space is abundant, people are more in need of venues suitable for socializing [8]. In addition, under previous development directions for urbanization, governments invested unequal amounts of resources in urban and rural area, causing differences in the living environment of these two types of area. For this reason, previous scholars employed the WHOQOL-BREF to measure the QoL of rural residents [52]. However, questionnaire distribution is limited by the characteristics of the surveyed population. For example, completing such comprehension-based questionnaires was relatively difficult for older adults, illiterate people, and people with low education attainment. Therefore, these scholars suggested including interviews in addition to the questionnaire for data collection [38].

The present study investigated a rural community in northeast China, where Chinese is the common language. A Chinese version of the WHOQOL-BREF was developed by other scholars, and all the items were verified to have adequate reliability and validity [16,51,53]. Therefore, this Chinese version was adopted for the survey in the present study. Although Chinese is widely used in China, the heterogeneity derived from geographical locations must be considered. Therefore, before the formal test was conducted, the questionnaire was administered to some of the participants for data collection and questionnaire review. This stage was referred to as the pretest, during which some rural residents and experts with relevant academic backgrounds were surveyed. The two tested groups are described in the following paragraphs.

For the experts, this study adopted a two-stage procedure for investigation. The first stage was rolling interviews, the purpose of which was to ensure the appropriateness of the items. The investigation was conducted by having the first expert revise the questionnaire, asking the second expert to provide comments on the revision results, and having the third and fourth experts reconfirm the results. The third and fourth experts did not change any items in the revised questionnaire, suggesting that the items had achieved convergence. A total of five experts whose academic backgrounds were rural community planning (*n* = 2), urban planning, landscape planning, and psychology, were interviewed in this stage. The experts generally approved of the basic framework of the indices and the investigation items; however, they indicated that the phrases commonly used by residents of northeastern rural area slightly differ from those of other regions. Therefore, the experts introduced local expressions into the questionnaire to enhance the comprehensibility of the items for the local residents. In addition, considering that the local residents tended to be relatively conservative, the experts suspected that the index of sexual behavior may be too direct to obtain a real assessment; thus, they suggested changing this item to a measure of spousal relationships. After criterion confirmation and item revision, the second stage involved using the importance level of the criteria to determine the importance of individual indices for QoL assessment. On a five-point Likert scale, the criteria were rated from 1 (very unimportant) to 5 (very important). The overall mean of the criteria was 4.17, indicating that the experts considered the indices as a whole achieved sufficient importance. Subsequently, the pretest was administered to local residents on the basis of this research framework.

**Figure 1:**
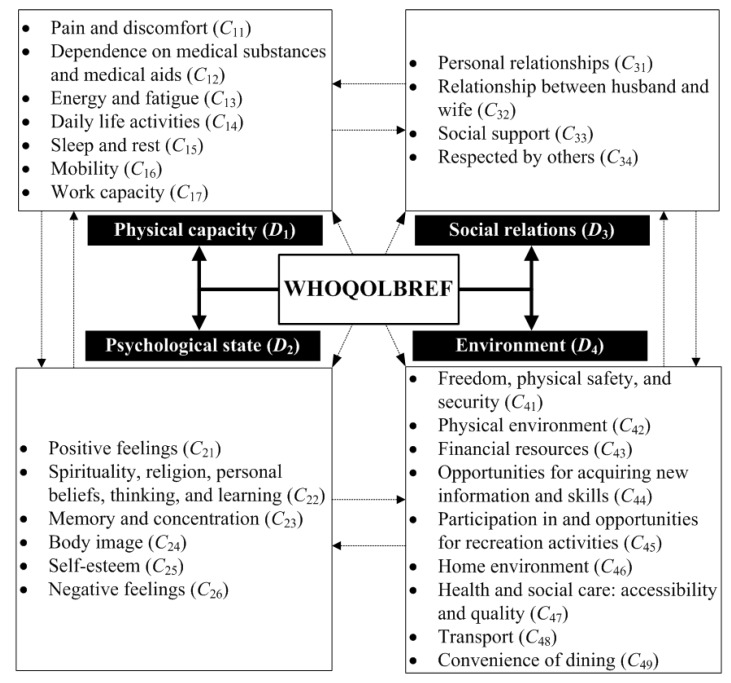
Indicator structure used in this study based on the Chinese version of WHOQOL-BREF (Brief version of quality of life framework proposed by the World Health Organization).

The revised questionnaire was distributed to residents of the investigated rural community for the pretest. In rural communities of northeast China, because of severe population aging, most residents are over 65 years old. Many of the older adults were illiterate; therefore, they could not complete the questionnaire by themselves. Although some young people have returned to Xingshisi Village for a living, most residents there were over 60 years old. Therefore, the research team conducted the investigation using a question and answer format. At first, these residents were relatively shy and reluctant to answer, fearing troubles that may arise from the investigation. They questioned our purposes repeatedly after every one or two questions. Nevertheless, they gradually relaxed during the survey process as our team members continually explained our purposes. At a later stage of investigation, the residents even aided us in questionnaire distribution. In this pretest, 31 copies of the questionnaire were distributed that contained three reverse-scored items. Because data collection was conducted through interviews as well as questionnaires, the results of positively and negatively worded items exhibited consistency. Therefore, all 31 copies were valid samples. The Cronbach’s α of the investigation results was 0.921, suggesting satisfactory reliability of the items. Thus, this index framework was adopted as a basis for subsequent investigations in this study. Figure 1 presents the index framework and the descriptions of the criteria are listed in Table A1.

## 3. The Modified DANP-mV Model

This section begins with the innovation of the DANP-mV model and subsequently explains the problems of the original model and improvements and advantages of the new model. Finally, the overall operation procedure and steps involved for the new model are described.

### 3.1. Innovation and Improvement of the DANP-mV Model 

The DANP-mV model uses a systematic method to propose an improved strategy based on the root cause of a problem [49]. Additionally, the model has evaluative and selective functions similar to those of traditional MADM methods to solve the selection problem of non-dominant solutions by using influential weights (IWs) [54,55]. Unlike the conventional method, DANP-mV puts more emphasis on a systematic way of looking at the problem. To utilize the output of this model, it is necessary to calculate the gap from the aspired level and the influential network relation map (INRM) [47]. 

Two novel approaches are involved in the DANP-mV model [18,47]. First is the calculation of the gap. This model believes that people naturally pursue an aspired level instead of an ideal point. There are unique results of using this type of gap. Decision makers can clearly recognize the gap for each criterion that needs improvement and the need for continuous improvement to achieve the aspired level. When evaluating a problem in practice, decision makers are sometimes faced with alternatives that are all good or all bad in a situation. In simple terms, if the bucket is full of rotten apples, no matter the choice, picking a better apple is still a rotten apple. To avoid this problem, decision makers can use this gap to identify situations in which all alternatives at each criterion are all good or bad. The second unique concept of this model explains how to systematically develop strategies. After realizing the problem, decision makers must develop improvement strategies to reach the goal. Traditionally, the model assumes that factors are independent of each other. Without interdependence between factors, decision makers can only think of ways to make improvements based on the obvious problem rather than addressing the root causes of the problem. However, in the real world, problems often involve a large and complex system. In this system, many factors have a mutual relationship that affects the goal. Therefore, this model is based on the root causes of the problem and discussed how to improve to achieve goals. This is what we call the systematic way of thinking in this research. Additionally, the INRM plays a central role in this study. The INRM is a map of interrelation between each factor. Unlike other display methods showing relationships based on the correlation coefficient, this map employs an arrow with directionality, and the direction of the arrow is determined by influence levels between the two criteria. Thus, after identifying which factor is problematic, decision makers can identify causal factors and build a comprehensive improvement strategy.

**Figure 2:**
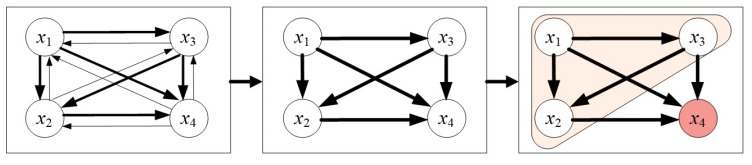
Development of the INRM (Influential Network Relation Map) and formulation of improvement strategies.

It is essential to determine whether a correlation exists between variables, but it is not very useful for decision makers to know the correlation between independent variables because decision makers want to know where the problems are, why they are problems, and how to solve them. If decision makers want to find the cause of problems, it is essential to identify which factors influence the occurrence of problems. There are ways to find the effects of these relationships, such as through setting thresholds. As previously described, in the DANP-mV model, the intensity of an influence is used to determine the effects of all the criteria. For example, assume again the four independent variables x_1_, x_2_, x_3_, and x_4_. As mentioned before, mutual influence relationships are present between the variables. Thus, there is a mutual influence between two criteria; x_1_ can affect x_2_, and x_2_ can influence x_1_. However, the effect of this relationship is not the same as its intensity. The thickness of the line represents the effect within the graphics; thicker lines indicate stronger effects. In this case, we assume that the relationship of x_1_ affecting x_2_, x_3_, and x_4_ is stronger than the relationship of x_2_, x_3_, and x_4_ affecting x_1_, and the relationship of x_3_ affecting x_2_ and x_4_ has a stronger effect than the relationship of x_2_ and x_4_ affecting x_3_, and so forth. Thus, the direction of influence is decided in this way. Finally, we determine that x_4_ is the problem point because x4 has the lowest performance and the highest gap. Using this system, decision makers can find causes and formulate improvement strategies from the source in a holistic manner, as shown in Figure 2.

Although the traditional DANP-mV model has many advantages, it also has a significant drawback; that is, it struggles to handle problems with numerous items. Although the method itself is powerful, if there is a problem with input data, the result of the calculation will be problematic. For example, if the overall structure has seven indicators, an item has 42 questions (7 × 6); if there are ten indicators, the item has 90 questions (10 × 9). When studying a practical issue, the indicators often exceed 15; therefore, the total number of items can exceed 210 questions (15 × 14). In addition, questionnaires rely heavily on the opinions of experts with professional qualities. Because of numerous problems, even experts require considerable time to complete the questionnaire. However, the willingness of time experts to commit to assist in research investigations is limited. Therefore, this study addresses this issue of the DANP-mV model and proposes a new version of the modified DANP-mV model. Then, this model is used to analyze empirical cases.

The modified DANP-mV model is intended to maintain the effect of the original model but address the problem of numerous items. The output of the original model has three results: the INRM, the IWs, and the identified criterion of poor performance. The modified DANP-mV model maintains these three results, but the differences lie in the illustration of the INRM and the calculation of IWs. 

We first explain differences in the INRM. When drawing the INRM for the original model, mutual influence between two criteria is represented by a line. However, in practice, numerous criteria lead to the complexity of the line graph and ultimately cause decision makers to be unable to clearly identify relationships from the INRM. Thus, past scholars have shown that the INRM of all criteria can be divided into two levels: dimensions and criteria. The dimension level shows the influences between each dimension, and the criteria level shows the influences of the criteria within the dimension. Therefore, interpreting the management implications requires explaining the influence relationships of the dimensions first and those of the criteria within the dimension second. When using the modified model in the data survey, we do not first obtain the effect relationship values of all criteria and then separate them into two levels. Instead, we directly divide the influences of dimensions and criteria at the beginning of the survey. In this way, if the indicator structure is composed of three dimensions and each dimension contains five criteria (total 15 criteria) and each dimension has six question items (3 × 2), the criteria comprise 60 items (5 × 4 + 5 × 4 + 5 × 4). The total number of questions is reduced from the original 210 items to 66 items. Next, the differences between IWs are described. When the weights are obtained, the original model first obtains the global weight of each criterion and then calculates the local weight of each dimension and criterion. The new model does this in the reverse order. We first obtain the local weights of dimensions and criteria within the dimension and subsequently calculate the global weight of each criterion. 

In addition to the advantage of the dramatic reduction of the number of items, the new model has characteristics that more closely approximate a real environment. Organizational operations in practice mostly use a hierarchical management structure, especially large enterprises. Each department has different businesses and responsibilities, and cross-sector business typically involves only middle and high-level management, whereas employees in lower positions are often only responsible for internal affairs of the department. Therefore, the new model has characteristics closer to those of practical problems.

### 3.2. Procedure of Modified DANP-mV Model 

The DANP-mV model is a hybrid research tool that consists of two technologies: DANP and modified VIKOR [54,55,56,57]. This method mainly uses questionnaires to collect data and establish a database. Questionnaires contain two parts: degree of influence between factors and degree of satisfaction of each indicator. Determining the degree of influence between factors relies on experts with relevant professional experience on the subject. Therefore, the respondents are limited. Depending on the topic, determining the satisfaction degree can employ experts or the public.

**Figure 3:**
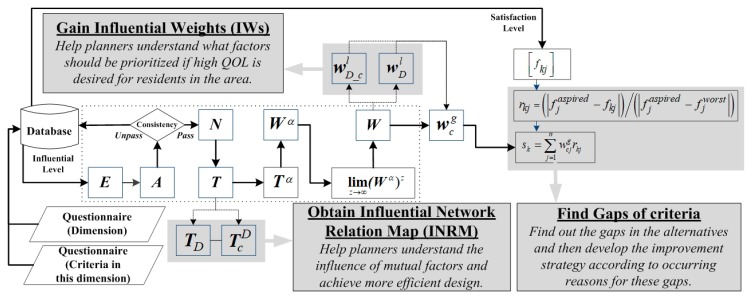
Procedure of the modified DANP-mV model.

Because the new model is based on this version and modified, it is called the modified DANP-mV model. Its operation is divided into 12 steps. In terms of the direct-influence relation matrix, the questionnaire design of the new model is different from that of the previous version. The new version is divided into dimensions and criteria within dimensions to collect data. Therefore, the operations of the first to eighth steps are respectively divided into these two parts for calculation. Thus, the local weights of dimensions and criteria within dimensions based on the DANP technique can be gained. “Factor” collectively describes both the dimensions and criteria in the following steps because the calculation procedure is the same. The global weight of all criteria can be obtained through the ninth step. Researchers can use this value to understand cross-dimensional information. The INRM can be drawn according to the sixth step, and the gaps of each alternative can be obtained through the tenth to twelfth steps. The entire process is shown in Figure 3.

**Figure 4:**
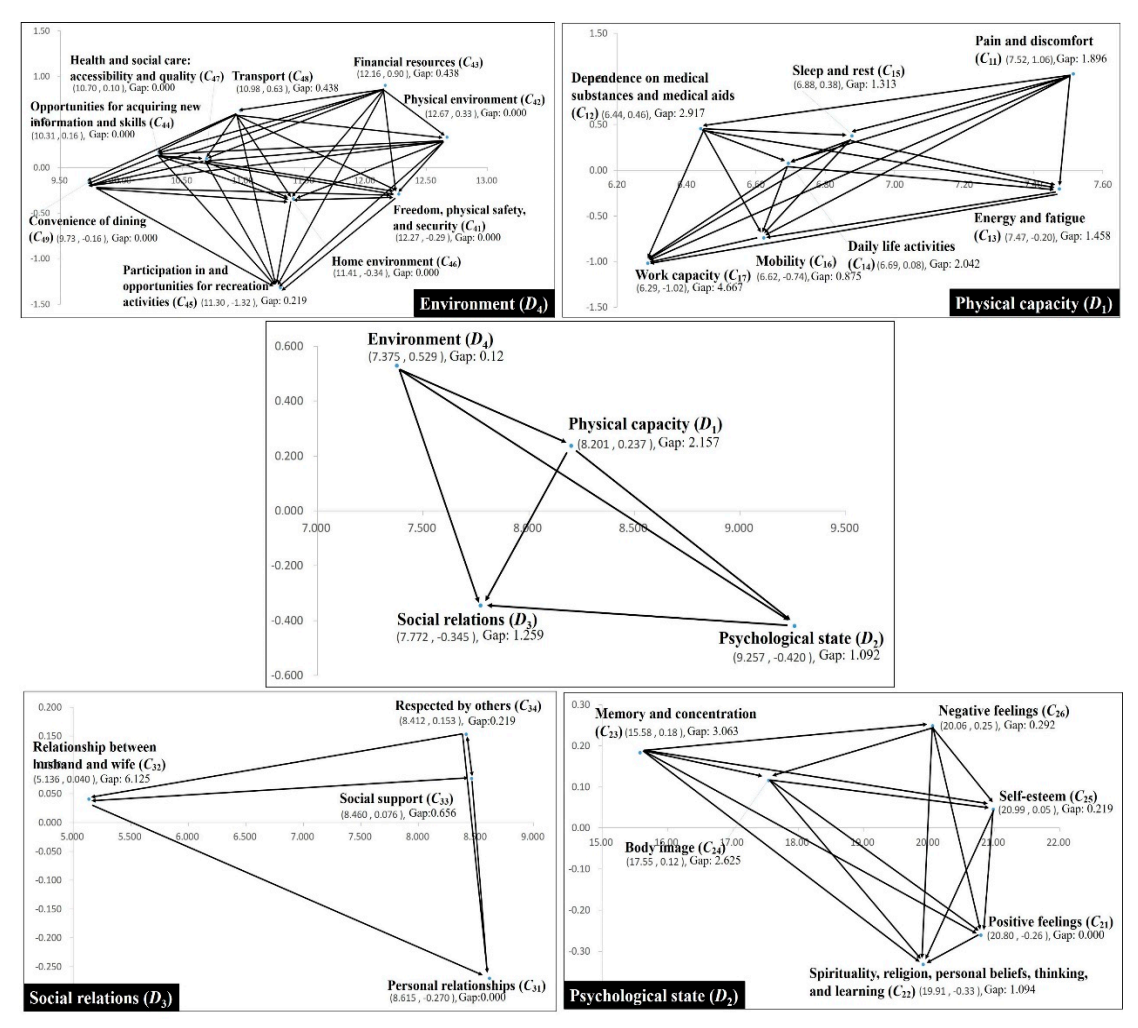
Overall INRM (influential network relation map).

In the following description of the method steps, in brief, the reader can regard formula 1 to formula 11 as a module (the module is shown in Figure 4 as a dotted box), After learning the influence of the dimension through the questionnaire, dimension weight can be calculated by using this module. Similarly, after obtaining the impact relationship of the criteria through the questionnaire, the weight of the criteria can be calculated by using this module.

*Step 1*: *Build the individual direct relation matrix **E***

The direct relation matrix is established through a pairwise comparison. Because the degree of influence between two factors is not necessarily the same, there is no reciprocal relationship between the pairwise comparisons of the matrix. The scale of the questionnaire uses an integer score of 0, 1, 2, 3, or 4, expressing the range from absolutely no influence (0) to very high influence (4) through natural language in linguistics [58]. Thus, this matrix is an *n × n* nonnegative matrix. Then, the direct-influence relation matrix of the *H* experts can be obtained through the questionnaire as shown in Equation (1). It can be expressed as ***E****^h^* = [*e*_*ij*_*^h^*]*_n_*_×*n*_ for *h* = 1, 2, …, *H*:

(1) $$E=\left[\begin{array}{ccccc} e_{11} & \cdots & e_{1j} & \cdots & e_{1n}\\ \vdots & & \vdots & & \vdots \\ e_{i1} & \cdots & e_{ij} & \cdots & e_{in}\\ \vdots & & \vdots & & \vdots \\ e_{n1} & \cdots & e_{nj} & \cdots & e_{nn} \end{array}\right]$$

*Step 2*: *Calculate the average direct-influence relation matrix **A***

The average scores of the *H* experts are $a_{ij}=\frac{1}{H}\sum_{h=1}^{H}e_{ij}^{h}$. The average matrix is called the average direct-influence relation matrix ***A***, and the degree of influence it receives from other factors is given by Equation (2):

(2) $$A=\left[\begin{array}{ccccc} a_{11} & \cdots & a_{1j} & \cdots & a_{1n}\\ \vdots & & \vdots & & \vdots \\ a_{i1} & \cdots & a_{ij} & \cdots & a_{in}\\ \vdots & & \vdots & & \vdots \\ a_{n1} & \cdots & a_{nj} & \cdots & a_{nn} \end{array}\right]$$

*Step 3*: *Examination of consistency*

The consistency value can be calculated according to Equation (3), which represents the consensus of the experts. It verifies whether the overall system has reached stability. The threshold for the value is 5%. If the value is less than 5%, confidence reaches more than 95%, meaning that the system has stabilized. However, if the value is greater than 5%, the system is not yet stable, and we must return to the first phase to determine whether the data collection is correct and whether the number of experts is sufficient:

(3) $$consistency=\frac{1}{n(n-1)}\sum_{i=1}^{n}\sum_{j=1}^{n}\left(\left|a_{ij}^{H}-a_{ij}^{H-1}\right|/a_{ij}^{H}\right)\times 100\%$$

*Step 4*: *Obtain the normalized average direct-influence relation matrix **N***

The normalized average direct-influence relation matrix ***N*** is acquired by normalizing matrix ***A***. Matrix ***N*** is easily derived from Equations (4) and (5), whereby all principal diagonal factors are equal to zero [59,60]:

(4) N=u⋅A

(5) $$\min\left\{\frac{1}{\max_{1\;\leq i\leq n}\sum_{j=1}^{n}a_{ij}},\frac{1}{\max_{1\;\leq j\leq n}\sum_{i=1}^{n}a_{ij}}\right\}$$

*Step 5*: *Obtain the total influence relationship matrix **T***

A continuous decrease of the indirect effects of problems moves with the powers of the matrix ***N****^q^* = [0]*_n_*_×*n*_ for $\lim_{q\to \infty }(I+N+N^{2}+\ldots +N^{q})=$(***I*** − ***N***)^−1^, where ***I*** is an *n × n* matrix [61]. The total influence relation matrix ***T*** is an *n × n* matrix, and is defined by ***T*** = [*t_ij_*]*_n×n_* for *i*,*j* = 1, 2, …, *K*, …, *n*, as shown in Equation (6):

(6) $$T=N+N^{2}+\ldots +N^{q}=N(I+N\;+\;N^{2}+\ldots +N^{q-1})=N(I+\;N\;+\;N^{2}+\ldots +N^{q-1})(I-N){\left(I-N\right)}^{-1}\;=N{\left(I-N\right)}^{-1},\;\mathrm{when}\;\lim_{q\to \infty }\;N^{q}={\left[0\right]}_{n\times n}$$

*Step 6*: *Illustrate the total INRM from the INRM of dimensions and criteria*

The total influence relation matrix ***T*** of the INRM can be acquired using Equations (7) and (8) to generate each row sum and column sum, respectively, in the matrix ***T***:

(7) $$o={\left(o_{i}\right)}_{n\times 1}={\left[\sum_{j=1}^{n}t_{ij}\right]}_{n\times 1}={\left(o_{1},\ldots ,o_{i},\ldots ,o_{n}\right)}^{\prime }$$

(8) $$r={\left(r_{j}\right)}_{n\times 1}={\left(r_{j}\right)}_{1\times n}^{\prime }={\left[\sum_{i=1}^{n}t_{ij}\right]}_{1\times n}^{\prime }={\left(r_{1},\ldots ,r_{j},\ldots ,r_{n}\right)}^{\prime },$$

where ***o****_i_* is the sum of a row in the total influence relation matrix ***T*** and represents the total effects (both direct and indirect) of factor *i* on the all other factors ${\left[\sum_{j=1}^{n}t_{ij}\right]}_{n\times 1}$. Similarly, *r_j_* is the column sum in the total influence relation matrix ***T*** and represents the total effects of factor *j* received from all other factors ${\left[\sum_{i=1}^{n}t_{ij}\right]}^{\prime }{}_{1\times n}$. The term (*o_i_* + *r_i_*) offers an index of the strength of the total influences given and received; that is, (*o_i_* + *r_i_*) indicates the degree of importance that factor *i* plays in the system. In addition, (*o_i_* − *r_i_*) provides an index of the degree of the cause of total influence. If (*o_i_* − *r_i_*) is positive, then criterion/perspective *i* is a net causer, and if (*o_i_* − *r_i_*) is negative, then factor *i* is a net receiver. Finally, drawing the INRM uses (*o_i_* − *r_i_*) as a y axis and (*o_i_* + *r_i_*) as an x axis to illustrate the scatter plot [62]. The party with a stronger influence relationship indicates the direction of the arrows between all influence factors. For instance, the influence of the first factor on the second factor is greater than the effect of the second factor on the first factor; therefore, the direction of the arrow is from the first factor to the second factor.

Because the new method investigates the dimensions and criteria separately during the questionnaire survey phase, we can directly obtain the total influence relation matrix of dimensions and criteria through the calculations in the first through sixth steps. Here, ***T****_D_* is the total influence relation matrix of dimensions, and ***T***_c_*^D^* is the total influence relation matrix of the criteria belonging to this dimension. The complete INRM integrates the INRMs of dimensions and criteria.

*Step 7*: *Transpose and normalize the total influence relation matrix*

Use Equation (9) to normalize the total influence relation matrix ***T***; then, the normalized total influence relation matrix ***T****^α^* can be calculated. Subsequently, the transpose matrix ***T****^α^* and the transposed total influence relation matrix ***w****^α^* can be obtained as shown in Equation (10):

(9) $$T^{\alpha }={\left[t_{ij}\right]}_{n\times n}/o$$

(10) $$W^{\alpha }={\left(T^{\alpha }\right)}^{-1}$$

*Step 8*: *Obtain local weights for dimensions w^l^_d_ and criteria w^l^_d_c_*

Limit the transposed total influence relation matrix ***w****^α^* by raising it to the *z* power until the ***w****^α^* has converged and become a stable matrix, in which *z* represents any number of power as shown in Equation (11). The local weights of dimension ***w****^l^_d_* and criteria ***w****^l^_d_c_* within the dimension are obtained; these are called the IWs in the DANP model:

(11) $$W=\underset{z\to \infty }{\lim}{\left(W^{\alpha }\right)}^{z}$$

*Step 9*: *Find global weights of all criteria*$W_{c}^{g}$

Global weights of all criteria $W_{c}^{g}$ are obtained by integrating the local weights of dimensions with criteria, as shown in Equation (12).

(12) $$W_{c}^{g}=W_{D\_c}^{l}\times W_{D}^{l}$$

*Step 10*: *Derive the aspiration levels and worst value*

Define the best value of *n* criteria, called the aspiration level, shown as *f*^aspired^ and the worst value shown as *f*^worst^. The aspiration level refers to the upper bound of the semantic scale in the questionnaire. By contrast, the worst case refers to the lower limit of the semantic scale in the questionnaire. In this study, the performance ranges from 0 to 10; 0 indicates very bad, and 10 indicates very good. These are used with natural language in the linguistic questionnaire. Thus, *f*^aspired^ is 10, and *f*^worst^ is 0.

*Step 11*: *Normalize performance of k alternatives, and calculate the gap*

The performance values of the *j* factors of the *k* alternatives are normalized, and the distance between these performance values and aspiration level is calculated at the same time. These normalized distances are gaps represented by *r_kj_*, as shown in Equation (13):

(13) $$r_{kj}=(|f_{}^{aspired}-f_{kj}|)/(|f_{}^{aspired}-f_{}^{worst}|)$$

*Step 12*: *Determine the mean group utility S_k_ for the gap*

Traditional VIKOR considers two types of gap: mean group utility and maximum regret degree. Because the modified DANP-mV model focuses on improving the performance of alternatives to achieve the aspiration level, the gap of this model adopts mean group utility *S_k_*. This value can be calculated using Equation (14):

(14) $$s_{k}=\sum_{j=1}^{n}w_{cj}^{g}r_{kj}$$

## 4. Improvement Strategy for QoL in Xingshisi Village

By using the Modified DANP-mV model, Xingshisi Village was analyzed; an improvement strategy based on the interaction was proposed to enable residents of the village to improve their QoL. This section is divided into three sections: Section 4.1 describes the background of Xingshisi Village. Section 4.2 details on data collection and analysis. Section 4.3 proposes an improvement strategy by discussing.

### 4.1. Introduction to Xingshisi Village

Xingshisi Village is located in Gannan County, Qiqihar City, Heilongjiang Province. The village has grown from 198 households and 956 rural people to 35 enterprises, with more than 1800 employees, and more than 1 billion yuan in total assets. The village uses the concept of “ideal village” for general planning. The village has facilities, such as schools, kindergartens, hospitals, facilities for the elderly, and public service systems, as well as complete infrastructure for water and sewage pipelines, water purification and sewage treatment plants, cable television, telephone, fiber optics, and refuse transfer. More notable are the large-scale forested green space and intelligent facilities that generate green energy. With the support of high-tech equipment, the village aims to develop high-value agricultural products. It also uses greenhouses, organic farming, and other cultivation methods. It has a product processing plant that can move products from primary to secondary production. With the support of relevant research units, industries in the village have been transformed into high-tech industries, such as the biomedicine industry. Only 2% of villagers are engaged in planting activities; the remaining 98% are involved in secondary and tertiary industries. This is different from most rural areas, where cereals are the main crops. Thanks to the favorable environment and support of related industries, many of the younger generation who originally worked in the city are willing to return to the village, making Xingshisi Village an ideal model of “production-life-ecology”. 

Xingshisi Village residents are mainly elderly people aged more than 60 years. Many of them have chronic diseases, such as cardiovascular or joint deterioration, causing them to require medical support. This rural area has the same characteristics as other rural areas; it has highly developed social networks. Residents in the village know each other and have a care system for the elderly. If there is a problem with the residents, not only the staff of the village care system but also neighbors or close friends come to assist. It is a community of strong mutual help. In general, residents sleep for 4–5 h and wake up naturally, feeling in good spirits. Most residents like to gather in the public open space of the village to chat or play mahjong in the community club. Most of them are optimistic and satisfied with their living conditions. Sleeping time is fixed. Most residents can handle daily affairs while paying attention to their own image and worrying less about their safety and financial situation. Because the area surrounding the village is farmland, connection with other areas has a certain isolation effect. In addition to regular buses, young people usually have their own cars for transportation, but residents rarely go to other towns unless they have important events.

### 4.2. Data Collection, Analysis, and Results

Data were collected from two parties: experts and residents of rural areas. Because it requires a certain degree of professionalism for assessment, the part on the degree of influence between the factors affecting QoL was answered by experts. Ten experts were surveyed. Of them, three were planners from the Urban Planning Institute who were mainly people responsible for the overall planning of Xingshisi and three were professors who had long been concerned with rural planning. Because the WHOQOL-BREF involves health-related issues, two experts were physicians from public hospitals. The remaining two experts were the director and deputy director of the village, responsible for follow-up maintenance of the overall rural environment, industrial operations, and resident care. They two experts were also residents of Xingshisi. At the time of the survey, interviews were also conducted. In addition to helping them understand the questions more comprehensively, we asked the experts to explain the reasons for the degree of influence. The experts were asked to provide an example, and each expert was administered a survey and interview. The time for interview was approximately 1.5 h. 

The second part of the analysis was the satisfaction of each indicator, using a Likert-type scale (7 points, 7 indicating “extreme satisfaction” and 1 indicating “extreme dissatisfaction”). In addition to experts living in the village, to avoid cognitive perception bias between other experts and local residents, the main respondents were rural residents. In this study, a total of 56 people were surveyed, a majority of whom were elderly people aged more than 60 years. Therefore, the direct use of questionnaire directly for the survey was not possible; hence, a question–answer method was used to data collection.

The calculation method was based on the steps of the modified DANP-mV model, and the operation results are shown in Table 1, Table 2, Table 3, Table 4 and Table 5. Table A1 lists the influence of the dimensions. The left panel shows the average direct-influence relationship matrix of the 10 experts. The consistency value was 0.024 (<0.05), indicating that the experts had reached a certain consensus overall. The overall system reached convergence. The right panel lists total influence relationship matrix obtained after calculation. Table 3, Table 4, Table 5 and Table 6 list the influence relationships of various criteria under each dimension. Each average direct-influence relationship matrix passed the consistency test. 

The integrated INRM is presented by first drawing the INRM of the dimension and the INRM of each criterion under each dimension and then summarizing it as shown in Figure 4. To achieve adequate QoL for residents, environment (*D*_4_) and physical ability (*D*_1_) were influential factors on the whole system, whereas psychological state (*D*_2_) and social relations (*D*_3_) were affected factors. 

Because each factor has an interaction relationship, when devising an improvement strategy, decision-makers should not focus only on the dimensions of the problem. More attention should be focused on the effects of environmental and physical capabilities. From the environment (*D*_4_) dimension, financial resources (*C*_43_), transport (*C*_48_), physical environment (*C*_42_), opportunities for acquiring new information and skills (*C*_44_), and health and social care: accessibility and quality (*C*_47_) were the affecting criteria, whereas convenience of dining (*C*_49_), home environment (*C*_46_), freedom, physical safety, and security (*C*_41_), and participation in and opportunities for recreation activities (*C*_45_) were the affected criteria. Similarly, because of the interplay between the criteria, if a decision-maker wants to effectively improve the performance of the environment (*D*_4_), they should consider financial resources (*C*_43_), transport (*C*_48_), physical environment (*C*_42_), opportunities for acquiring new information and skills (*C*_44_), and health and social care (*C*_47_) on the system. The physical capacity (*D*_1_) dimension was similar; if this dimension is to be effectively promoted, pain and discomfort (*C*_11_), sleep and rest (*C*_15_), dependence on medical substances and medical aids (*C*_12_), and daily life activities (*C*_14_) should be the focus. Respect from others (*C*_34_), social support (*C*_33_), and relationship between husband and wife (*C*_32_) are influential in the system of social relations (*D*_3_). Finally, within psychological state (*D*_2_), negative feelings (*C*_26_), memory and concentration (*C*_23_), and self-esteem (*C*_25_) were the criteria for influence.

The results of IWs and Gap of each factor are shown in Table 6. At first, The local weight of the psychological state (*D*_2_) was 0.295; that is, if the rural villagers want high QoL, they must prioritize psychological state (*D*_2_), followed by social relations (*D*_3_), physical capacity (*D*_1_), and environment (*D*_4_). The overall performance was 5.811. The following criteria all showed full marks: positive feelings (*C*_21_); personal relationships (*C*_31_); freedom, physical safety, and security (*C*_41_); physical environment (*C*_42_); opportunities for acquiring new information and skills (*C*_44_); home environment (*C*_46_); health and social care: accessibility and quality (*C*_47_); and convenience of dining (*C*_49_). Most full marks belonged to the environment (*D*_4_) dimension. This also means that the villagers had a certain degree of recognition of the environment. However, these criteria naturally exhibited no gap from the aspiration level.

Second, In general, the largest dimension by gap value was physical capacity (*D*_1_), which was 2.157. The criterion for the largest gap in physical capacity (*D*_1_) was work capacity (*C*_17_), which had a value of 4.667. The criterion for the largest gap value in psychological state (*D*_2_) was memory and concentration (*C*_23_) at 3.063. The largest criterion for gap value in social relations (*D*_3_) was relationship between husband and wife (*C*_32_), which had a value of 6.125. This criterion was also the criterion with the highest gap among all criteria. The gap of the environment (*D*_4_) dimension was clearly lower than others, with a value of 0.12. Within this dimension, the criteria with the largest gap values were financial resources (*C*_43_) and transport (*C*_48_), which had values of 0.438.

### 4.3. Discussion and Proposed Improvement Strategy

From the performance levels of the overall indicator structure (dimensions and criteria) Xingshisi Village in Gannan County was a benchmark for rural areas in northeastern China, the QoL of the villagers is high. However, although the village is modeled as an ideal village, a gap exists between the villagers’ QoL and the ideal environment. This means that although there is no external paradigm, only room for continuous improvement exists. Although young villagers are willing to return to their hometowns, rural areas remain dominated by older people. Therefore, the gap of the physical capacity (*D*_1_) was largest. As people age, the ability to rely on physical activity becomes relatively limited. Therefore, the largest gap in that dimension was work capacity (*C*_17_). It is also because of the increase in age that memory and mental concentration begin to be affected. During the interview, many interviewees said that they often forget things and that their spirit is not as concentrated as before. Thus, memory and concentration (*C*_23_) in the psychological state (*D*_2_) dimension had a high gap. Relationship between husband and wife (*C*_32_) remained to demonstrate highest gap among all criteria because most respondents said their spouse had died and they did not consider looking for another spouse. Finally, the villagers were more satisfied with the environmental performance, but still hoped to effectively improve the financial resources (*C*_43_) and transport (*C*_48_) criteria. 

The improvement strategy of this study is divided into dimensions and criteria for discussion. On the dimension level, the villagers hope to improve physical capacity (*D*_1_). Through the dimension section of the INRM (Figure 4), it can be found that physical capacity (*D*_1_) is affected by the environment (*D*_4_). The problem of physical ability is often caused by aging. If decision-makers focus only on improving problems, they may not be able to produce certain results. Therefore, the dimension level suggests that decision-makers consider the effects of environmental changes on physical capacity. Supplementing or providing improved medical facilities and health care systems is an effective way to improve village health and QoL. 

In the physical capacity (*D*_1_) dimension, what the villagers hoped to improve was work capacity (*C*_17_), but work capacity is subject to pain and discomfort (*C*_11_), sleep and rest (*C*_15_), dependence on medical substances and aids (*C*_12_), and daily life activities (*C*_14_). Ijn other words, policymakers should start considering how to improve the health of villagers rather than how to improve their ability to work. Therefore, reducing pain and increasing medical care, thereby improving sleep quality and gradually developing work ability through daily activities, is necessary. This is the direction for improvement in the physical capacity (*D*_1_) dimension.

The problem with the psychological state (*D*_2_) dimension is in the memory and concentration (*C*_23_) criterion, but this criterion belongs to the source of the influence; therefore, policymakers must more consider the effects of the environment and physical capacity on this criterion. Within the social relations (*D*_3_) dimension, villagers hope to improve the relationship between husband and wife (*C*_32_). This criterion is influenced by two criteria: respect of others (*C*_34_) and social support (*C*_33_). This means that the improvement direction of this criterion should be to increase the respect and support of respondents from others and strengthen social harmony and inclusiveness, deepen contact between villagers of different ages, and allow older people to live with dignity. Financial resources (*C*_43_) and transport (*C*_48_) in the environment (*D*_4_) dimension are criteria requiring improvement. These criteria are the source of influence for this dimension. Simultaneously, this dimension is the source of influence for the entire system. Therefore, overall, the improvement of Xingshisi Village should first improve protection of the villagers’ financial income and increase related social welfare measures as well as medical care facilities and services. After the villagers’ financial income is more effectively protected, the villagers can perform other related leisure activities. By improving social welfare measures and medical facilities and services, villagers can remain healthy and avoid pain. Having a healthy body can bring positive emotions, reduce negative thinking and improve mental state for villagers. Finally, by increasing inclusiveness, older individuals can have positive interactions with other groups, creating a more harmonious rural atmosphere and achieve the goal of a higher QoL for Xingshisi Village.

This study has three limitations. First, this study was a case study specific to Xingshisi Village; however, methods should vary according to the heterogeneity of each place and the time at which the study is performed. Therefore, although the finding of this study can be used as a reference for rural development of other villages, the characteristics of heterogeneity and timeliness must be considered. Second, at the time of the survey, some interviewees said that they had not left the rural area to visit other larger rural areas or cites; therefore, some questionnaires may have presented the related biases. In future, researchers should include the experts to centerfire this bias. Third, although the village is experiencing a return of the youth population, aging remains a common phenomenon. Therefore, in future, researchers should use the WHOQOL-OLD questionnaire if the surveyed villagers are mostly elderly people [63].

## 5. Conclusions 

This study had three main findings: (1) Xingshisi Village, Gannan County, Qiqihar City is located in northeastern China. It is a model village built based on the ideal village. For this reason, this study used the village as the research object. The results of the study showed that the villagers’ satisfaction with the village was 5.811. This shows that although the village was built based on the ideal properties, QoL in this village exhibits a 1.189 gap requiring improvement. (2) The new modified model not only displays the original effect of the INRM and IWs but also reduced the total number of items from 650 (26 × 25) to 168 (4 × 3 + 7 × 6 + 6 × 5 + 4 × 3 + 9 × 8). (3) the largest gap was found in the dimension of physical capacity (*D*_1_). In other words, although the village has a young population, it still has aging-related problems. Although the current medical system can mitigate some of these problems, it cannot completely alleviate them. Whether such resource input improves the QoL of villagers required investigation. Proposing an improvement strategy from the source of the problem represents a novel rationale. The dimension of the INRM suggest that physical capacity (*D*_1_) is affected by the environment (*D*_4_). In other words, if managers want to improve QoL in their villages, they must be concerned about the effects of environmental change. This study aimed to improve the QoL of Xingshisi Village. The villagers’ financial income should be protected effectively, and relevant social welfare measures and medical care facilities and services should be added to keep the villagers healthy. This study believes that to improve QoL in Xingshisi Village, more effectively protecting villagers’ financial revenue, increasing relevant social welfare measures as well as medical facilities and services, and maintaining the health and lives of the villagers are essential. They should be assisted in avoiding disease, increasing positive emotions, reducing negative thoughts, and increasing inclusiveness to allow older individuals to interact with other groups to create a more harmonious rural atmosphere and improve livability in Xingshisi Village.

## Figures and Tables

**Table 1:** Average direct-influence relation matrix and total influence relation matrix of each dimension.

*A*	*D*_1_	*D*_2_	*D*_3_	*D*_4_	*T*	*D*_1_	*D*_2_	*D*_3_	*D*_4_
***D***_1_	0	3.500	2.200	2.000	***D***_1_	0.873	1.334	1.086	0.926
***D***_2_	2.900	0	3.300	2.100	***D***_2_	1.146	1.108	1.196	0.967
***D***_3_	1.700	3.000	0	1.900	***D***_3_	0.924	1.174	0.787	0.829
***D***_4_	2.600	2.800	1.700	0	***D***_4_	1.038	1.222	0.990	0.702

Note: Consistency value = 0.024 < 0.05.

**Table 2:** The Average-direct influence relation matrix and total influence relation matrix of criteria in ***D***_1_.

*A*	*C*_11_	*C*_12_	*C*_13_	*C*_14_	*C*_15_	*C*_16_	*C*_17_	*T*	*C*_11_	*C*_12_	*C*_13_	*C*_14_	*C*_15_	*C*_16_	*C*_17_
***C***_11_	0	3.500	3.500	3.100	3.000	3.000	3.200	***C***_11_	0.463	0.583	0.703	0.612	0.600	0.661	0.665
***C***_12_	3.000	0	2.800	2.200	2.300	2.300	2.300	***C***_12_	0.507	0.346	0.569	0.485	0.482	0.531	0.528
***C***_13_	2.600	2.000	0	2.800	2.700	3.100	2.900	***C***_13_	0.509	0.456	0.465	0.528	0.517	0.586	0.574
***C***_14_	2.200	1.900	2.800	0	1.900	3.100	3.100	***C***_14_	0.465	0.425	0.559	0.373	0.457	0.555	0.551
***C***_15_	2.700	2.500	3.100	2.400	0	2.600	2.600	***C***_15_	0.514	0.477	0.604	0.512	0.394	0.565	0.561
***C***_16_	1.900	1.500	2.600	2.300	2.300	0	2.000	***C***_16_	0.406	0.366	0.495	0.432	0.426	0.362	0.453
***C***_17_	1.700	1.600	2.300	1.600	1.900	2.100	0	***C***_17_	0.365	0.339	0.443	0.368	0.376	0.422	0.321

Note: Consistency value = 0.041 < 0.05.

**Table 3:** The Average-direct influence relation matrix and total influence relation matrix of criteria in ***D***_2_.

*A*	*C*_21_	*C*_22_	*C*_23_	*C*_24_	*C*_25_	*C*_26_	*T*	*C*_21_	*C*_22_	*C*_23_	*C*_24_	*C*_25_	*C*_26_
***C***_21_	0	3.100	2.400	2.800	3.500	3.000	***C***_21_	1.733	1.836	1.402	1.591	1.911	1.795
***C***_22_	3.200	0	2.200	2.100	3.300	3.100	***C***_22_	1.827	1.590	1.335	1.492	1.822	1.724
***C***_23_	2.300	2.300	0	1.500	2.200	2.600	***C***_23_	1.461	1.410	0.974	1.192	1.449	1.397
***C***_24_	2.800	2.600	1.500	0	3.000	2.400	***C***_24_	1.647	1.580	1.181	1.236	1.649	1.539
***C***_25_	3.500	3.500	2.200	2.900	0	3.100	***C***_25_	1.963	1.894	1.424	1.630	1.766	1.839
***C***_26_	3.400	3.000	2.300	2.800	3.100	0	***C***_26_	1.897	1.813	1.384	1.575	1.874	1.612

Note: Consistency value = 0.002 < 0.05.

**Table 4:** The Average-direct influence relation matrix and total influence relation matrix of criteria in ***D***_3_.

*A*	*C*_31_	*C*_32_	*C*_33_	*C*_34_	*T*	*C*_31_	*C*_32_	*C*_33_	*C*_34_
***C***_31_	0	1.700	3.300	2.900	***C***_31_	1.021	0.733	1.229	1.190
***C***_32_	1.900	0	1.300	1.400	***C***_32_	0.794	0.361	0.718	0.715
***C***_33_	3.300	1.300	0	3.400	***C***_33_	1.310	0.719	0.994	1.246
***C***_34_	3.400	1.500	3.200	0	***C***_34_	1.318	0.735	1.251	0.979

Note: Consistency value = 0.03 < 0.05.

**Table 5:** The Average-direct influence relation matrix and total influence relation matrix of criteria in ***D***_4_.

*A*	*C*_41_	*C*_42_	*C*_43_	*C*_44_	*C*_45_	*C*_46_	*C*_47_	*C*_48_	*C*_49_	*T*	*C*_41_	*C*_42_	*C*_43_	*C*_44_	*C*_45_	*C*_46_	*C*_47_	*C*_48_	*C*_49_
***C***_41_	0	2.700	3.000	1.600	2.800	2.600	2.500	1.700	1.500	***C***_41_	0.642	0.747	0.702	0.591	0.765	0.715	0.650	0.606	0.574
***C***_42_	2.900	0	1.700	2.300	2.900	3.000	2.600	2.700	2.400	***C***_42_	0.819	0.683	0.701	0.661	0.822	0.777	0.699	0.688	0.651
***C***_43_	2.800	2.500	0	2.400	2.900	3.000	2.100	2.400	2.400	***C***_43_	0.818	0.795	0.627	0.669	0.826	0.782	0.682	0.679	0.654
***C***_44_	2.000	1.900	2.200	0	2.500	2.100	1.900	1.900	1.500	***C***_44_	0.648	0.634	0.599	0.452	0.671	0.617	0.556	0.546	0.509
***C***_45_	1.700	2.300	2.200	1.800	0	2.100	1.500	1.800	1.700	***C***_45_	0.611	0.625	0.575	0.513	0.537	0.593	0.519	0.521	0.498
***C***_46_	2.800	3.000	2.200	1.900	2.200	0	1.700	1.500	1.500	***C***_46_	0.713	0.710	0.628	0.563	0.693	0.556	0.577	0.557	0.535
***C***_47_	2.800	2.200	1.800	1.700	1.900	1.900	0	2.300	1.900	***C***_47_	0.698	0.664	0.599	0.543	0.665	0.625	0.488	0.576	0.539
***C***_48_	2.600	2.500	2.200	2.100	2.300	1.800	2.200	0	2.200	***C***_48_	0.733	0.717	0.653	0.594	0.725	0.662	0.621	0.511	0.586
***C***_49_	2.000	2.000	2.000	1.700	2.100	1.500	1.700	1.500	0	***C***_49_	0.600	0.591	0.548	0.490	0.607	0.548	0.508	0.490	0.403

Note: Consistency value = 0.038 < 0.05.

**Table 6:** The performance and gap evaluation of the case study using DANP-mV model.

Dimensions/Criteria	Local Weight	Global Weight	Performance	Gap
Physical capacity (*D*_1_)	0.245		4.843	2.157
Pain and discomfort (*C*_11_)	0.136	0.033	5.104	1.896
Dependence on medical substances and medical aids (*C*_12_)	0.125	0.031	4.083	2.917
Energy and fatigue (*C*_13_)	0.160	0.039	5.542	1.458
Daily life activities (*C*_14_)	0.138	0.034	4.958	2.042
Sleep and rest (*C*_15_)	0.136	0.033	5.688	1.313
Mobility (*C*_16_)	0.153	0.037	6.125	0.875
Work capacity (*C*_17_)	0.151	0.037	2.333	4.667
Psychological state (*D*_2_)	0.295		5.908	1.092
Positive feelings (*C*_21_)	0.183	0.054	7.000	0.000
Spirituality, religion, personal beliefs, thinking, and learning (*C*_22_)	0.176	0.052	5.906	1.094
Memory and concentration (*C*_23_)	0.134	0.040	3.938	3.063
Body image (*C*_24_)	0.152	0.045	4.375	2.625
Self-esteem (*C*_25_)	0.182	0.054	6.781	0.219
Negative feelings (*C*_26_)	0.173	0.051	6.708	0.292
Social relations (*D*_3_)	0.249		5.741	1.259
Personal relationships (*C*_31_)	0.289	0.072	7.000	0.000
Relationship between husband and wife (*C*_32_)	0.167	0.041	0.875	6.125
Social support (*C*_33_)	0.274	0.068	6.344	0.656
Respected by others (*C*_34_)	0.270	0.067	6.781	0.219
Environment (*D*_4_)	0.211		6.880	0.120
Freedom, physical safety, and security (*C*_41_)	0.124	0.026	7.000	0.000
Physical environment (*C*_42_)	0.122	0.026	7.000	0.000
Financial resources (*C*_43_)	0.111	0.024	6.563	0.438
Opportunities for acquiring new information and skills (*C*_44_)	0.100	0.021	7.000	0.000
Participation in and opportunities for recreation activities (*C*_45_)	0.124	0.026	6.781	0.219
Home environment (*C*_46_)	0.116	0.024	7.000	0.000
Health and social care: accessibility and quality (*C*_47_)	0.104	0.022	7.000	0.000
Transport (*C*_48_)	0.102	0.022	6.563	0.438
Convenience of dining (*C*_49_)	0.097	0.021	7.000	0.000
Total Performance			5.811	
Total Gap				1.189

## Appendix A

The indicators in this study were based on the Chinese version of WHOQOL-BREF, which is different from the original version in that the Chinese version is particular to the Chinese region and has two additional indicators: Transport (*C*_48_) and Convenience of dining (*C*_49_). In this study, some names were adjusted and defined for the characteristics of rural residents. The results are shown in Table A1.

**Table A1:** Description of all criteria in the Chinese version of WHOQOL framework.

Criterion Name	Description
Pain and discomfort (*C*_11_)	Measures whether the respondents have a physical state of illness.
Dependence on medical substances and medical aids (*C*_12_)	Indicates the status of respondents with chronic diseases.
Energy and fatigue (*C*_13_)	Determines the respondents’ vitality.
Daily life activities (*C*_14_)	Determines the respondents’ ability to care for themselves.
Sleep and rest (*C*_15_)	Surveys the respondents’ quality of sleep and rest.
Mobility (*C*_16_)	Determines the respondents’ mobility.
Work capacity (*C*_17_)	Indicates the respondents’ ability to work.
Positive feelings (*C*_21_)	Determines the respondents’ positive emotional state.
Spirituality, religion, personal beliefs, thinking, and learning (*C*_22_)	Indicates the respondents’ state of belief.
Memory and concentration (*C*_23_)	Determines the respondents’ memory and concentration.
Body image (*C*_24_)	Refers to the respondents’ external feelings.
Self-esteem (*C*_25_)	Refers to respondents’ cognitive evaluation of self.
Negative feelings (*C*_26_)	Determines the respondents’ negative emotional state.
Personal relationships (*C*_31_)	Refers to the relationship between respondents and their families.
Relationship between husband and wife (*C*_32_)	Refers to the feelings between husband and wife.
Social support (*C*_33_)	
Respected by others (*C*_34_)	Refers to the relationship between other people and the respondents.
Freedom, physical safety, and security (*C*_41_)	Refers to the respondents’ safe and free living environment.
Physical environment (*C*_42_)	Refers to the respondents’ external living environment.
Financial resources (*C*_43_)	Mainly refers to the respondents’ financial status.
Opportunities for acquiring new information and skills (*C*_44_)	Mainly refers to whether the respondents’ environment facilitates gaining of new knowledge and skills.
Participation in and opportunities for recreation activities (*C*_45_)	Mainly refers to whether the respondents’ environment has related entertainment activities.
Home environment (*C*_46_)	Refers to the respondents’ internal living environment.
Health and social care: accessibility and quality (*C*_47_)	Indicates whether the respondents’ living environment has adequate health care services.
Transport (*C*_48_)	Refers to the degree of traffic convenience of the respondents’ environment.
Convenience of dining (*C*_49_)	Determines the respondents’ dietary environment.
